# Early Stages of Obesity-related Heart Failure Are Associated with Natriuretic Peptide Deficiency and an Overall Lack of Neurohormonal Activation: The Copenhagen Heart Failure Risk Study

**DOI:** 10.5334/gh.776

**Published:** 2020-03-25

**Authors:** Freja Stoltze Gaborit, Caroline Kistorp, Thomas Kümler, Christian Hassager, Niels Tønder, Kasper Iversen, Pia R. Kamstrup, Jens Faber, Lars Køber, Morten Schou

**Affiliations:** 1 Department of Cardiology, Herlev Gentofte University Hospital, DK; 2 Department Endocrinology, Rigshospitalet, Copenhagen, DK; 3 Department of Cardiology, Rigshospitalet, Copenhagen, DK; 4 Department of Cardiology, North Zealand University Hospital, DK; 5 Department of Clinical Biochemistry, Herlev Gentofte University Hospital, DK; 6 Department Endocrinology, Herlev Gentofte University Hospital, DK; 7 Department of Clinical Medicine, University of Copenhagen, DK; 8 Faculty of Health and Medical Science, University of Copenhagen, DK

**Keywords:** obesity, natriuretic peptide, heart failure, neurohormonal activation

## Abstract

**Objective:**

This study evaluated the associations between the natriuretic peptide activity and the neurohormonal response in non-obese and obese outpatients with and without heart failure (HF).

**Background:**

Obesity-related HF may be a distinct subtype of HF. Obesity is associated with lower plasma concentrations of natriuretic peptides. The associations between obesity and neurohormonal activation estimated by mid-regional pro-adrenomedullin (MR-proADM) and copeptin in patients with HF is not elucidated.

**Methods:**

This prospective cohort-study included 392 outpatients ≥60years, plus ≥1 risk-factor(-s) for HF (hypertension, ischemic heart disease, atrial fibrillation, diabetes, chronic kidney disease), and without known HF. Patients were categorized ‘non-obese’ BMI = 18.5–29.9 kg/m^2^ (n = 273) and ‘obese’ BMI ≥ 30 kg/m^2^ (n = 119). The diagnosis of HF required signs, symptoms, and abnormal echocardiography. NT-proBNP, MR-proANP, MR-proADM, and copeptin were analyzed.

**Results:**

Obese patients were younger, had a higher prevalence of diabetes and chronic kidney disease, but a lower prevalence of atrial fibrillation. A total of 39 (14.3%) non-obese and 26 (21.8%) obese patients were diagnosed with HF. In obese patients, HF was not associated with higher plasma concentrations of NT-proBNP (Estimate: 0.063; 95%CI: –0.037–1.300; P = 0.064), MR-proANP (Estimate: 0.207; 95%CI: –0.101–0.515; P = 0.187), MR-proADM (Estimate: 0.112; 95%CI: –0.047–0.271; P = 0.168), or copeptin (Estimate: 0.093; 95%CI: –0.333–0.518; P = 0.669). Additionally, obese patients with HF had lower plasma concentrations of NT-proBNP (Estimate: –0.998; 95%CI: –1.778–0.218; P = 0.012), and MR-proANP (Estimate: –0.488; 95%CI: –0.845–0.132; P = 0.007) compared to non-obese patients with HF, whereas plasma concentrations of MR-proADM (Estimate: 0.066; 95%CI: –0.119–0.250; P = 0.484) and copeptin (Estimate: 0.140; 95%CI: –0.354–0.633; P = 0.578) were comparable.

**Conclusions:**

Patients with obesity-related HF have natriuretic peptide deficiency and lack of increased plasma concentrations of MR-proADM and copeptin suggesting that patients with obesity-related HF have a blunted overall neurohormonal activity.

## Introduction

The global epidemic of obesity is an increasing burden of risk for HF [1,2]. Obesity is a well-established risk factor for HF, independent of obesity-related conditions like hypertension, type 2 diabetes, and ischemic heart disease [3]. Obesity has also been associated with subclinical changes in the structure and function of the left ventricle (LV), especially changes of the diastolic function and LV hypertrophy [4,5]. Thus, obesity has a strong correlation with HF with preserved ejection fraction (HFpEF) [6]. Various hypotheses of the pathophysiological mechanisms for obesity-related HF have been proposed: volume overload and increased afterload leading to hypertrophy of the LV [7], lipotoxicity caused by increased circulating free fatty acids [8], altered metabolism in the myocardium due to insulin resistance [9], and activation of the leptin-aldosterone-neprilysin axis leading to sodium retention, plasma volume expansion and ventricular remodelling [10].

Furthermore, obesity has been associated with lower plasma concentrations of amino-terminal pro-B-type natriuretic peptide (NT-proBNP) and mid-regional pro-atrial natriuretic peptide (MR-proANP) [11,12,13], a condition described as natriuretic peptide deficiency. But the mechanisms behind and the implications of the natriuretic peptide deficiency are incompletely understood. Structural remodelling of the heart in obese patients and the type of HF may influence the secretion of the natriuretic peptides since HFpEF is associated with lower plasma concentrations of the natriuretic peptides than HFrEF [14,15]. Increased epicardial fat in obese patients with HFpEF has been suggested to reduce the LV wall stress due to an enhanced pericardial resistance and thus impair the stimuli of the natriuretic peptides synthesis [16]. Metabolic factors may also influence the plasma concentrations of the natriuretic peptides, e.g. insulin-resistance has been associated with an augmented elimination of the natriuretic peptides from the circulation [17]. Considering that blockade or modulation of the neurohormonal activation is a cornerstone in the recommended treatment of patients with HFrEF [18] and is under evaluation in patients with HFpEF, obesity-related HF may be a subtype of HF, that due to natriuretic peptide deficiency, would benefit from a diverse management e.g. early treatment with sacubitril-valsartan. It is unknown whether obesity-related HF is associated with lower plasma concentrations of novel cardiac biomarkers like mid-regional pro-adrenomedullin (MR-proADM) and copeptin reflecting neurohormonal activation.

Accordingly, we evaluated the plasma concentrations of natriuretic peptides and neurohormonal activation estimated by copeptin and MR-proADM in elderly non-obese and obese outpatients, with and without HF. Further, to improve the understanding of echocardiographic findings in obesity-related HF we assessed the relationship between plasma concentrations of natriuretic peptides and important echocardiographic parameters stratified according to the presence or absence of obesity.

## Methods

The Copenhagen Heart Failure Risk Study was a prospective cohort study evaluating the prevalence of early stages of HF and undiagnosed HF among elderly outpatients with a high risk of HF but without known or suspected HF [19].

### Study population

The inclusion and exclusion criteria have been described in detail previously [19]. Briefly, patients were eligible if they were 60 years of age or above, had one or more risk factor(s) for HF (hypertension, ischemic heart disease, atrial fibrillation, diabetes, stroke, chronic kidney disease). Main exclusion criterions were: a history of HF or reduced LVEF, suspected HF, ongoing advanced cardiac disease (including known moderate-severe valvular disease), recent or planned cardiac procedure, moderate-severe chronic obstructive pulmonary disease, estimated glomerular filtrations rate (eGFR)<15 ml/min/1.73 m^2^. Enrolment commenced in December 2014 and ended in June 2017. Patients were consecutively screened at the Department of Cardiology, the Clinic of Diabetes, and the Clinic of Nephrology, Herlev and Gentofte University Hospital, Denmark, eligible patients were included after discharge from the Department of Cardiology or after a scheduled visit at the outpatient clinic of Diabetes or at the Clinic of Nephrology. In total 400 patients were enrolled in the Copenhagen Heart Failure Risk Study. Echocardiography was missing in one patient, who was therefore excluded from analysis. Further, in this present study, we excluded patients with BMI < 18.5 kg/m^2^ (n = 7 patients), resulting in a study population of 392 patients.

Written informed consent was obtained from all patients prior to enrolment. The Committee on Health Research Ethics for the Capital Region of Denmark approved the study (H-3-2014-016). The study was conducted according to the Declaration of Helsinki.

Physical examination was performed at the day of enrolment, this included a medical history, current medication, blood pressure, venous blood samples and echocardiogram. Patients’ weight and height were registered. BMI was calculated: weight (kg) divided by height squared (m^2^). Patients were categorized as ‘obese’ (BMI ≥ 30 kg/m^2^) and ‘non-obese’ (BMI ≥ 18.5 to < 30.0 kg/m^2^). The diagnosis of HF was evaluated according to the 2012 guidelines on HF form the European Society of Cardiology [20], which includes clinical signs of HF, symptoms of HF and specific echocardiographic abnormalities. The 2016 guidelines on HF from the European Society of Cardiology [18], have incorporated plasma concentrations of BNP/NT-proBNP in the diagnosis of both HFpEF and HFrEF. Since this study investigated the influence of obesity on plasma NT-proBNP concentrations and other cardiac biomarkers we used the 2012 definition of HF. Patients with symptoms of HF, clinical signs of HF and abnormal echocardiography were diagnosed with HF. Symptoms of HF were patient-reported, and we required a combination of minimum two symptoms: a) dyspnoea/orthopnoea, b) edema/treatment with loop diuretics. Clinical signs of HF at physical examination: peripheral edema, observed/confirmed dyspnea (New York Heart Association [NYHA] functional class II–IV), neck-vein dilation, third heart-sound, pulmonary crepitations. Structural or functional abnormalities in the heart were evaluated with echocardiography. Abnormal echocardiography, study definition (one criteria): LVEF: Female ≤ 53%, Male ≤ 51%; LV end-diastolic volume (LVEDV) indexed to body surface area (BSA): Female > 61 ml/m^2^, Male > 74 ml/m^2^; LV mass indexed to BSA: Female > 96 g/m^2^, Male > 116 g/m^2^; e’septal: Female < 4.1 cm/sec, Male < 4.3 cm/sec; lateral E/e′ > 13, septal E/e′ > 15; left atrial volume indexed to BSA ≥ 35 ml/m^2^; global longitudinal strain (GLS): Female > –15.3%, Male > –14.7%; moderate-severe aortic stenosis (aortic valvular area < 1cm^2^), moderate-severe aortic regurgitation, severe mitral stenosis. In addition, LVEF ≤ 40% were defined as HF, even if the patient did not present with symptoms or signs of HF.

### Biomarkers

Biochemical analyses were performed at the local laboratory. Routine biomarkers were analysed using fresh samples: haemoglobin, creatinine, cholesterol, high-sensitive C-reactive peptide, haemoglobin A1C, alanine aminotransferase, alkali phosphatase. Likewise using fresh samples, plasma concentrations of NT-proBNP (ng/L) were measured using the two-site chemiluminescent immunometric assay, IMMULITE2000 NT-proBNP (Siemens Healthcare Diagnostics). The analytical sensitivity of the assay reported by the manufacturer was 10 pg/mL, the reportable range 20–35000 pg/mL. The precision has been tested and for a mean plasma concentration 145–8884 pg/mL the within-run coefficient of variance was 2.3–3.2%, and the total coefficient of variance was 4.0%–4.9% according to the manufacturer, confirmed in a multicentre evaluation [21]. Blood for subsequent analyses was centrifuged for 10 minutes at 3000g and 20°Celsius, plasma was frozen and stored at –80°Celsius. Plasma concentrations of MR-proANP (pmol/L) were measured using an automated immunofluorescent assay, BRAHMS MR-proANP KRYPTOR (Thermo Fisher Scientific), assay information was provided by the manufacturer. The limit of quantitation was 4.5 pmol/L and the precision for plasma concentrations between 20–1000 pmol/L with an intra assay coefficient of variance <2.5% and an inter assay coefficient of variance <6.5%, analytical performance has been tested in healthy individuals [22]. Plasma concentrations of MR-proADM (nmol/L) and copeptin (C-terminal-pro-vasopressin)(pmol/L) were measured using specified automated immunofluorescent assays, BRAHMS KRYPTOR (Thermo Fisher Scientific) [23,24]. The limit of quantification for the MR-proADM assay has been reported as 0.23nmol/L and the precision for plasma concentrations between 0.2–6.0nmol/L with an intra assay coefficient of variance ≤10% and an inter assay coefficient of variance ≤11%, with the highest coefficient of variance for plasma concentrations between 0.2–0.5nmol/L. The limit of detection for the copeptin assay has been reported as 0.69 pmol/L and the precision for plasma concentrations 2.0 –> 50 pmol/L with an intra assay coefficient of variance <15% and an inter assay coefficient of variance <18%, with the highest coefficient for plasma concentrations below 4 pmol/L. Assay information was provided by the manufacturer.

### Echocardiography

Image acquisition was performed according to a predefined study protocol using the Vivid-E9 ultrasound system (General Electric Vingmed Ultrasound, Horten, Norway). Analyses were performed using the EchoPAC (version 201,70.1, General Electric Vingmed Ultrasound, Horten, Norway). Analyses were performed off-line, and in accordance with current guidelines [25,26], by a trained physician, blinded to clinical and biochemical data.

Dimensions of the LV were obtained from the parasternal long-axis view. Inter-ventricular septal thickness, LV internal diameter, and LV posterior wall thickness were measured at end-diastole and LV mass was calculated. LV mass indexed to BSA may underestimate hypertrophy in obese patients, therefore, we calculated LV mass indexed to BSA and to height [27]. The apical 4- and 2-chamber view were used to assess LVEDV and LVEF. We used the Simpson’s biplane method to calculate LVEF, however, in 19 patients this was not possible, instead, LVEF was calculated using the Teichholz’s method. Wall motion score was evaluated in the three apical views, using the 16-segments model. Left atrial volume was assessed by planimetric in the apical 2- and 4-chamber view. The diastolic function was evaluated using pulse wave Doppler at the mitral inflow (peak early = E; atrial = A) and mitral valve deceleration time, and with tissue Doppler for myocardial velocities septal and lateral (peak early = e’; peak systolic = s’). The right ventricle function was evaluated by tricuspid annular plane systolic excursion at the lateral tricuspid annulus. Longitudinal strain was obtained in the three apical views, and global longitudinal strain (GLS) was calculated. Circumferential strain was obtained in the parasternal short-axis view at the papillary muscle level.

### Statistics

Baseline characteristics and echocardiographic parameters are presented in four groups: non-obese patients with and without HF, and obese patients with and without HF. Categorical variables as numbers of patients and percentages, compared using the chi-square test. Continuous variables as median with 25- and 75-percentile, mean ± standard derivation (SD) and compared using the Wilcoxon test. To account for skewness continuous variables were log_2_ transformed for comparison if appropriate. Obese and non-obese patients with HF were compared, and obese and non-obese patients without HF were compared, respectively.

To evaluate the association between obesity and HF we created three logistic regression models. The outcome variable was HF. Model 1 included obesity, age, and sex. Model 2 included variables from model 1 plus atrial fibrillation, hypertension, ischemic heart disease, diabetes, stroke, eGFR < 60 ml/min/1.73 m^2^. Model 3 included variables from model 2 plus HDL-cholesterol (Female > 1.2 mmol/L, Male > 1.0 mmol/L), LDL cholesterol (<2.6 mmol/L), Lipoprotein(a) (>50 mg/dL).

The relationship between obesity, the diagnosis of HF, and cardiac biomarkers were evaluated with linear regression models. Interaction analysis showed significant interaction between HF and obesity, this interaction was therefore included in the linear regression models. Cardiac biomarkers were log_2_-transformed. Comparisons were made with two-way ANOVA analysis, adjusted for age, sex, eGFR.

The diagnostic ability of the cardia biomarkers for diagnosis of HF in non-obese and obese patients, respectively, was conducted using Receiver Operating Characteristics curves (ROC), and area under the curve (AUC) was calculated.

The influence of obesity on the association between plasma concentrations of NT-proBNP or MR-proANP and selected echocardiographic parameters were evaluated using linear regression models. Echocardiographic parameters were response variables. Plasma concentrations of NT-proBNP and MR-proANP were log_2_-transformed. The model was adjusted for age, gender, eGFR, and atrial fibrillation. Each model was tested for interaction between obesity and plasma concentrations of NT-proBNP or MR-proANP, respectively.

The diagnostic findings in obese and non-obese patients were evaluated as the prevalence of HF, the prevalence of patient-reported symptoms of HF, the prevalence of clinical signs of HF, and the prevalence of abnormal echocardiography, respectively. Trend tests were performed using the Cochran Armitage trend test.

For all statistical analyses, the level of significance was set to P < 0.05 (two-sided). Analyses were performed using SAS enterprise, version 7.11 (SAS Institute Inc., Cary, NC, USA).

## Results

### Study population

In total 119 patients were categorized ‘obese’ and 273 patients were ‘non-obese’. Median age was 72 years, and 48% were female. The prevalence of undiagnosed HF was comparable among non-obese and obese patients, in total 39 of the non-obese patients (14.3%) and 26 of the obese patients (21.8%) were diagnosed with HF according to the diagnostic criteria for HF (P = 0.076 for comparison). The baseline characteristics are presented in Table 1. In general, clinical and biochemical characteristics were similar between the obese and the non-obese patients with HF. However, obese patients with HF were younger than non-obese patients with HF and had a higher prevalence of diabetes, but a lower prevalence of atrial fibrillation.

**Table 1:** **Baseline characteristics for patients categorized as ‘Non-obese’ and ‘Obese’ according to HF status.** Data are presented as median (25-, 75-percentile), and n (%).

Variable	Non-obese (n = 273)		Obese (n = 119)	

	Without HF (n = 234)	With HF (n = 39)	Without HF (n = 93)	With HF (n = 26)

**Age, years**	72 (68, 78)	80 (71, 85)	69 (65, 74)*	71 (68, 74)†
**Female, female**	107 (45.7%)	25 (64.1%)	44 (47.3%)	14 (53.6%)
**Systolic blood pressure, mmHg**	138 (127, 149)	138 (125, 153)	133 (125, 145)	135 (125, 145)
**Diastolic blood pressure, mmHg**	79 (73, 86)	75 (66, 85)	80 (74, 85)	78 (72, 86)
**Heart rate, beats/min**	68 (60, 76)	68 (60, 80)	74 (61, 83)*	72 (68, 79)
**BMI**	25.8 (23.5, 27.6)	26.1 (23.4, 28.4)	32.3 (31.3, 34.6)*	35.6 (32.6, 38.7)†
**Smoking current or past, %**	140 (59.8%)	8 (34,8%)	62 (66.7%)	18 (42.9%)
**NYHA class I, n (%)**	170 (72.7%)	7 (18.0%)	52 (55.9%)*	3 (11.5%)
**NYHA class II, n (%)**	55 (23.5%)	25 (64.1%)	39 (41.9%)*	22 (84.6%)†
**NYHA class III, n (%)**	9 (3.9%)	7 (18.0%)	2 (2.2%)	1 (3.9)
**Framingham 5-year HF risk, score**	10 (8, 14)	12 (10, 16)	11 (8,15)	14 (10,16)
**Minnesota LWHFQ, score**	9 (2,22)	24 (14, 41)	13 (2,36)	32 (14, 54)
**Inclusion site and indication for hospitalization**				
**Department of Cardiology, n, (%)**	155 (66.2%)	29 (74.4%)	41 (44.1%)*	16 (61.5%)
**Chest pain, n (%)**	78 (33.3%)	9 (23.1%)	19 (20.4%)	9 (30.8%)
**Palpitations/arrhythmia, n (%)**	46 (19.7%)	13 (33.3%)	15 (16.1%)	3 (11.5%)
**Syncope, n (%)**	20 (8.6%)	5 (12.8%)	4 (4.3%)	0
**Dyspnea, n (%)**	4 (1.7%)	2 (5.1%)	1 (1.1%)	5 (19.2%)
**Other, n (%)**	7 (3.0%)	0	2 (2.2%)	0
**Outpatient Clinic of Diabetes, n (%)**	40 (17.1%)	4 (10.3%)	24 25.8%)	8 (30.8%)†
**Outpatient Clinic of Nephrology, n (%)**	39 (16.7%)	6 (15.4%)	28 (30.1%)*	2 (7.7%)
**Medical history**				
**Hypertension, %**	189 (80.8%)	33 (84.6%)	81 (87.1%)	22 (84.6%)
**Ischemic heart disease, %**	62 (26.5%)	13 (33.3%)	17 (18.3%)	6 (23.1%)
**Atrial fibrillation, %**	68 (29.1%)	23 (59.0%)	19 (20.4%)	5 (19.2%)†
**Diabetes, %**	71 (30.3%)	7 (18.0%)	48 (51.6%)*	15 (57.7%)†
**Chronic kidney disease, %**	34 (14.5%)	3 (7.7%)	21 (22.6%)	5 (19.2)
**Stroke, %**	25 (10.7%)	7 (18.0%)	13 (14.0%)	2 (7.7%)
**Medication**				
**ACE inhibitor, n (%)**	67 (28.6%)	7 (18.0%)	27 (29.0%)	4 (15.4%)
**Angiotensin II-receptor-antagonist, n (%)**	74 (31.6%)	12 (30.8%)	37 (39.8%)	15 (57.7%)†
**Aldosterone antagonist, n (%)**	2 (0.9%)	2 (5.1%)	1 (1.1%)	0
**Calcium antagonist, n (%)**	72 (30.8%)	12 (30.8%)	31 (33.3%)	8 (30.1%)
**Beta blocker, n (%)**	102 (43.6%)	25 (64.1%)	44 (47.3%)	12 (46.2%)
**Loop diuretics, n (%)**	18 (7.7%)	15 (38.5%)	19 (20.4%)*	7 (26.9%)
**Thiazide, n (%)**	74 (31.6%)	5 (12.8%)	36 (38.7%)	9 (34.6%)†
**Statin, n (%)**	143 (61.1%)	23 (59.0%)	64 (68.8%)	15 (57.7%)
**Per oral antidiabetics, n (%)**	56 (23.9%)	7 (18.0%)	38 (40.9%)*	10 (38.5%)
**Insulin, n (%)**	30 (12.8%)	3 (7.7%)	23 (24.7%)*	7 (26.9%)†
**Biochemistry**				
**Haemoglobin, mmol/L**	8.6 (8.0, 9.1)	8.2 (7.9, 8.8)	8.6 (8.2, 9.2)	8.4 (7.6, 8.8)
**CRP ≥3 mg/L, %**	66 (28.2%)	12 (30.8%)	33 (35.5%)	14 (53.9%)
**HbA1c, mmol/mol**	39 (37, 44)	39 (35, 45)	45 (38, 58)*	43 (39, 57)†
**Creatinine**, µ**mol/L**	81 (70, 100)	88 (68, 118)	87 (70, 121)	84 (74, 109)
**eGFR, mL/min/1.73m**^2^	72 (55,87)	59 (43, 81)	71 (45, 87)	75 (51, 86)
**Alkali phosphatase, U/l**	66 (56, 79)	72 (65, 92)	72 (60, 84)*	81 (64, 90)
**Total cholesterol, mmol/L**	4.4 (3.8, 5.3)	4.5 (3.5, 5.2)	4.3 (3.7, 5.2)	4.3 (3.5, 4.9)
**HDL, mmol/L**	1.30 (1.05, 1.62)	1.35 (1.07, 1.74)	1.15 (0.97, 1.36)*	1.09 (0.95, 1.46)†
**LDL, mmol/L**	2.2 (1.7, 3.0)	2.20 (1.70, 2.70)	2.1 (1.5, 2.7)*	1.85 (1.50, 2.70)
**Troponin I ≥10ng/L**	48 (20.6%)	17 (43.6%)	8 (8.7%)*	5 (19.2%)†
**NT-proBNP, pg/mL**	202 (99, 523)	1050 (221, 3040)	120 (73, 339)*	196 (113, 740)†
**NT-proBNP > 125**	158 (67.5%)	35 (89.7%)	45 (48.4%)*	19 (73.1%)
**MR-proANP, pmol/L**	130.4 (89.9, 192.9)	246.3 (143.5, 341.5)	100.1 (70.9, 147.7)*	117.2 (71.3, 201.4)†
**MR-proADM, nmol/L**	0.77 (0.61, 0.99)	1.04 (0.86, 1.22)	0.88 (0.70, 1.18)*	0.94 (0.80, 1.12)
**Copeptin, pmol/L**	6.1 (3.8, 12.2)	7.4 (5.4, 20.6)	8.8 (5.3, 16.8)*	10.2 (5.4, 17.2)

* Indicates significant difference (p < 0.05) among patients without HF ‘Non-obese’ vs ‘Obese’. † Indicates significant difference (p < 0.05) among patients with HF ‘Non-obese’ vs ‘Obese’. Abbreviations: HF, heart failure; BMI, body mass index; NYHA, New York heart association; LWHFQ, living with heart failure questionnaire; ACE, angiotensin-converting-enzyme; CRP, c-reactive protein; HbA1c, haemoglobin 1Ac; eGFR, estimated glomerular filtration rate; HDL, high-density lipoprotein; LDL, low-density lipoprotein; NT-proBNP, amino-terminal pro-B-type-natriuretic-peptide, MR-proANP, midregional pro-atrial-natriuretic-peptide; MR-proADM, midregional-pro-adrenomedullin.

As expected, plasma concentrations of NT-proBNP were higher in patients with HF, compared to patients without HF in both non-obese and obese patients (median: 202 vs. 1050 ng/L and 120 vs. 196 ng/L; mean ± SD: 479 ± 698 vs. 2334 ng/L ± 4060 and 259 ± 305 vs. 805 ng/L ± 1981, respectively, all P < 0.001 and P = 0.047). The plasma concentrations of MR-proANP were higher in non-obese patients with HF, compared to patients without HF (median: 130 vs. 246 pmol/L; mean ± SD: 153 ± 95 vs. 271 pmol/L ± 169; P < 0.001), but not in obese patients with HF (median: 100 vs. 117 pmol/L; mean ±: 121 ± 74 vs. 151 pmol/L ± 106; P = 0.2137). Similar pattern for plasma concentrations of MR-proADM (median: 0.77 vs. 1.04nmol/L and 0.88 vs. 0.94nmol/L; mean ± SD: 0.84 ± 0.31 vs. 1.10nmol/L ± 0.43 and 0.98 ± 0.39 vs. 1.01nmol/L ± 0.34; P < 0.001 and P = 0.395). As well as for plasma concentrations of copeptin in non-obese and obese patients, respectively, (median: 6.1 vs. 7.4 pmol/L and 8.8 vs. 10.2 pmol/L; mean ± SD: 10.4 ± 11.9 vs. 15.2 pmol/L ± 17.8 and 13.3 ± 11.7 vs. 12.9 pmol/L ± 11.2, P = 0.027 and P = 0.974).

The echocardiographic parameters are presented in Table 2. No major differences were encountered between obese patients with HF and non-obese patients with HF. The systolic function was largely preserved and the median of, LVEF in obese patients with HF was 55.7% and 61.2%, respectively, in non-obese patients (P = 0.668) There was no difference in the prevalence of the HF sub-types among the obese and the non-obese patients; in total 8 (2.9%) non-obese patients and 4 (3.3%) obese patients had HFrEF (LVEF < 40%), 5 (1.8%) non-obese patients and 2 (1.7%) obese patients had HFmrEF (LVEF 40–49%), and 26 (9.5%) non-obese patients and 20 (16.8%) obese patients had HFpEF (LVEF ≥ 50%), (P > 0.05 for all). The overall diastolic function was mildly abnormal when considering median values of all patients [26]. LAESV was comparable between obese and non-obese patients with HF (64.3 mL vs. 68.8 mL) (P = 0.583). However, LAESV indexed to BSA was higher in non-obese patients with HF (41.0 mL/m^2^ vs 32.8 mL/m^2^) (P = 0.036). LVEDV was higher among obese patients with HF compared to non-obese patients with HF (88.21 mL vs. 64.31 mL) (P = 0.003), this difference persisted after adjusting for height (P = 0.003), but not when adjusting for BSA (P = 0.069).

**Table 2:** Echocardiographic parameters for patients categorized as ‘Non-obese’ and ‘Obese’ according to HF status. Data are presented as median (25-, 75-percentile), and n (%).

Variable	Non-obese (n = 273)		Obese (n = 119)	

	Without HF (n = 234)	With HF (n = 39)	Without HF (n = 93)	With HF (n = 26)

**Systolic function**				
**LVEF, %**	62.9 (56.8, 68.0)	61.2 (43.8, 70.4)	60.7 (54.9, 66.8)	55.7 (50.1, 67.3)
**LVEF ≥50%**	224 (95.7%)	26 (66.7%)	82 (88.2%)*	20 (76.9%)
**LVEF 40–49%**	10 (4.3%)	5 (12.8%)	11 (11.8%)*	2 (7.7%)
**LVEF <40%**	–	8 (20.5%)	–	4 (15.4%)
**Wall motion score**	16 (16, 16)	16 (16, 20)	16 (16, 16)	16 (16, 17)
**S’ septal, cm/sec**	7.0 (6.0, 8.1)	6.0 (5.2, 7.0)	7.2 (6.3, 8.3)	7.0 (6.1, 7.7)†
**S’ lateral, cm/sec**	8.1 (7.0, 9.3)	6.3 (5.0, 8.5)	8.3 (7.0 (9.2)	7.4 (6.8, 8.7)†
**GLS, %**	–21.4 (–23.3, –18.7)	–20.7 (–24.2, –16.9)	–20.1 (–22.2, –17.8)*	–18.6 (–22.3, –14.8)
**CS, %**	–29.1 (–33.6, –25.2)	–26.8 (–32.5, –23.6)	–28.7 (–33.9, –23.9)	–26.8 (–31.0, –21.7)
**Diastolic function**				
**E’ septal, cm/sec**	6.14 (5.28, 7.75)	5.72 (4.65, 6.57)	6.90 (5.59, 7.91)	6.21 (5.13, 7.58)
**E/e’ septal, cm/sec**	11.2 (9.1, 13.7)	14.5 (11.7, 17.2)	11.2 (9.5, 13.5)	12.0 (10.2, 16.1)
**E/A**	0.87 (0.72, 1.02)	0.88 (0.74, 1.26)	0.85 (0.71, 1.03)	0.81 (0.73, 0.99)
**MV deceleration time, ms**	265 (222, 312)	252 (205, 333)	263 (223, 307)	266 (252, 300)
**LAESV, mL**	52.9 (40.5, 64.7)	68.8 (56.0, 83.2)	55.1 (46.5, 65.7)	64.3 (52.6, 89.3)
**LAESV indexed to BSA, mL/m**^2^	29.1 (24.2, 35.5)	41.0 (34.6, 49.3)	28.1 (23.9, 32.6)	32.8 (27.4, 45.2)†
**LAESV indexed to height, mL/m**	30.4 (23.7, 36.7)	40.7 (35.4, 48.9)	31.7 (27.4, 37.6)*	39.7 (29.7, 49.9)
**Structural Changes**				
**IVSd, cm**	0.93 (0.82, 1.02)	1.00 (0.80, 1.12)	0.97 (0.86, 1.08)*	1.00 (0.89, 1.11)
**LVIDd, cm**	4.69 (4.27, 5.11)	4.85 (4.20, 5.60)	4.88 (4.42, 5.36)	5.03 (4.64, 5.41)
**LV mass, g**	147,16 (118.05, 173.21)	163.34 (121.72, 224.14)	150 (125.45, 183.86)	185.74 (133.40, 212.76)
**LVmass indexed to BSA, g/m**^2^	76.03 (65.68, 87.76)	91.59 (70.60, 119.58)	72.68 (63.40, 86.96)	82.26 (68.63, 99.30)
**LVmass indexed to height, g/m**	83.74 (69.75, 98.50)	99.92 (69.95, 130.30)	87.74 (74.87, 104.56)*	108.80 (82.05, 120.89)
**LVEDV, mL**	72.29 (60.30, 94.89)	64.31 (57.93, 80.43)	82.03 (66.90, 97.41)*	88.21 (74.74, 110.69)†
**LVEDV indexed BSA, ml/m**^2^	39.69 (33.56, 48.88)	35.68 (31.28, 43.99)	39.32 (32.79, 47.45)	41.90 (35.42, 50.46)
**LVEDV indexed to height, ml/m**	42.73 (35.19, 53.72)	39.19 (34.31, 48.19)	47.94 (39.41, 56.72)*	52.43(43.35, 66.21)†
**Right ventricular function**				
**TAPSE, mm**	22.5 (19.6, 25.5)	20.1 (16.7, 23.3)	22.2 (20.4, 25.2)	21.1 (18.7, 23.1)

* Indicates significant difference (p < 0.05) among patients without HF ‘Non-obese’ vs ‘Obese’. † Indicates significant difference (p < 0.05) among patients with HF ‘Non-obese’ vs ‘Obese’. Abbreviations: LV = left ventricle; EF = ejection fraction; GLS = global longitudinal strain; CS = circumferential strain; LAESV = left atrial end systolic volume; BSA =body surface area; LVIDd = left ventricle inter dimensional diastole; LVEDV = left ventricle end diastolic volume; TAPSE = tricuspid annular plane systolic excursion.

### Diagnosis of HF and relation with obesity

Obesity was associated with HF in a multivariate logistic regression model (Table 3). In analogous analyses, with BMI categorized as normal weight, overweight and obese, the association remained significant for obese patients (OR 3.10; 95% CI 1.34–7.16; P = 0.003), but not for over-weight patients (OR 1.32; 95% CI 0.61–2.87; P = 0.350), adjusted for traditional risk factors (supplemental Table 1).

**Table 3:** Association between obesity and HF in logistic regression models.

	Odds Ratio 95% Confidence Interval	Estimate 95% Confidence Interval	p-value

Model 1			
**Obesity**	2.47 (1.34–4.55)	0.453 (0.148–0.758)	0.004
Model 2			
**Obesity**	2.62 (1.38–4.98)	0.482 (0.161–0.803)	0.003
Model 3			
**Obesity**	2.56 (1.35–4.89)	0.471 (0.148–0.793)	0.004

Model 1: Age and gender (reference female). Model 2: variables in model 1, Diabetes, Atrial fibrillation, Hypertension, Ischemic heart disease, eGFR <60 ml/min/1.73m^2^, Stroke. Model 3: variables in model 2, HDL-cholesterol (>1.2 mmol/L women and >1.0 mmol/L men), LDL cholesterol (<2.6 mmol/L), Lipoprotein A (>50 mg/dL).

**Figure 1:**
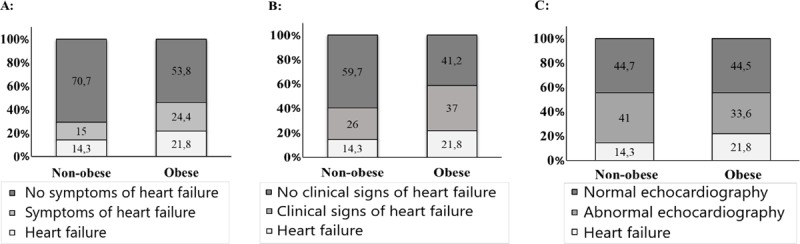
**Diagnostic findings in the non-obese and obese patients. (A)** Patient reported symptoms of heart failure (test for trend P = 0.004); **(B)** Clinical signs of heart failure, (test for trend P = 0.002); **(C)** Abnormal echocardiography (test for trend P = 0.336).

Among the obese patients 21.8% were diagnosed with HF, in addition, 24.4% of the obese patients reported symptoms of HF, 37.0% had clinical signs of HF, and 33.6% had an abnormal echocardiography without fulfilling the diagnostic criteria for HF (Figure 1). Among the non-obese patients 14.3% were diagnosed with HF. Among the non-obese patients without a diagnosis of HF, the prevalence of patient-reported symptoms of HF, or clinical signs of HF was lower compared to the obese patients, 15.0% and 26.0%, respectively (P-value for trend < 0.05 for both). The prevalence of an abnormal echocardiography among patients without a diagnosis of HF was comparable (non-obese: 41.0% and obese: 33.6%; P for trend = 0.336.

### Cardiac biomarkers and obesity

**Figure 2A–D:**
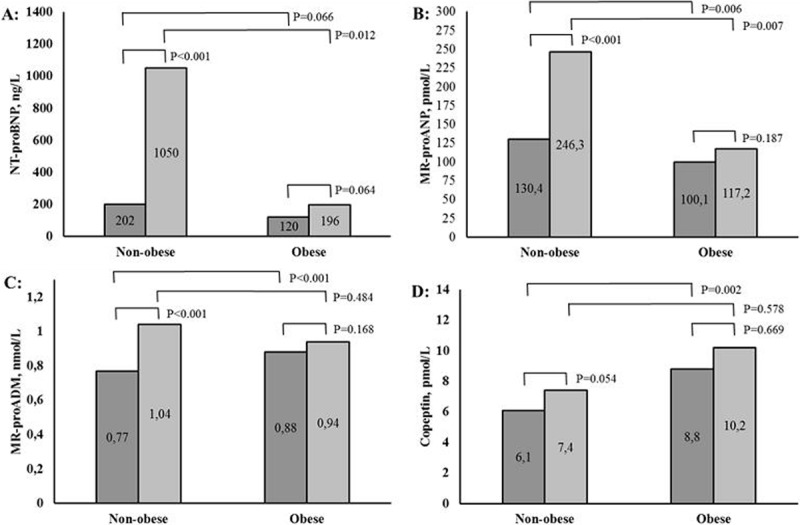
**Plasma concentrations of cardiac biomarkers for obese and non-obese patients, with and without HF**. Median values are presented in each box, and p-values represent comparison between categories adjusted for age, sex, eGFR.

Figure 2 A–D shows plasma concentrations of the cardiac biomarkers in patients with and without HF divided according to obesity. In obese patients, plasma concentrations of the cardiac biomarkers did not differ between patients with and without HF, NT-proBNP (Estimate: 0.063; T-value 1.84; 95%CI –0.037–1.300; P = 0.064), MR-proANP (Estimate: 0.207; T = 1.32; 95%CI –0.101–0.515; P = 0.187), MR-proADM (Estimate: 0.112; T = 1.38; 95%CI –0.047–0.271; P = 0.168), Copeptin (Estimate: 0.093; T = 0.43; 95%CI –0.333–0.518; P = 0.669). In non-obese patients, plasma concentrations of NT-proBNP (Estimate 1.271; T = 4.64; 95%CI 0.732–1.811; P < 0.001), MR-proANP (Estimate 0.453; T = 3.65; 95%CI 0.209–0.697; P < 0.001), and MR-proADM (Estimate 0.241; T = 3.76; 95%CI 0.115–0.367; P < 0.001) were higher in patients with HF, compared to patients without HF. Plasma concentrations of Copeptin were comparable in non-obese patients with and without HF (Estimate 0.332; T = 1.94; 95%CI -0.005–0.670; P = 0.054).

When considering plasma concentrations of the cardiac biomarkers in the patients with HF, plasma concentrations of NT-proBNP (Estimate: –0.998; T = –2.52; 95%CI –1.778–0.218; P = 0.012) and MR-proANP (Estimate: –0.488; T = –0.845–0.132; P = 0.007) were lower in obese patients, compared to non-obese patients, whereas no differences in plasma concentrations of MR-proADM (Estimate: 0.066; T = 0.70; 95%CI –0.119–0.250; P = 0.484) or copeptin (Estimate: 0.140; T = 0.56; 95%CI –0.354–0.633; P = 0.578) were observed. In patients without HF, plasma concentrations of NT-proBNP were comparable between obese and non-obese patients (Estimate: –0.358; T = –1.84; 95%CI –0.739–0.024; P = 0.066), whereas, plasma concentrations of MR-proANP were lower in obese patients (Estimate: –0.242; T = –2.74; 95%CI –0.415–0.069; P = 0.006), and plasma concentrations of MR-proADM (0.195; T = 4.28; 0.105; 95%CI 0.105–0.285; P < 0.001) and copeptin (Estimate: 0.380; T = 3.11; 95%CI 0.140–0.620; P = 0.002) were higher in obese patients (Figure 2A–D).

**Figure 3:**
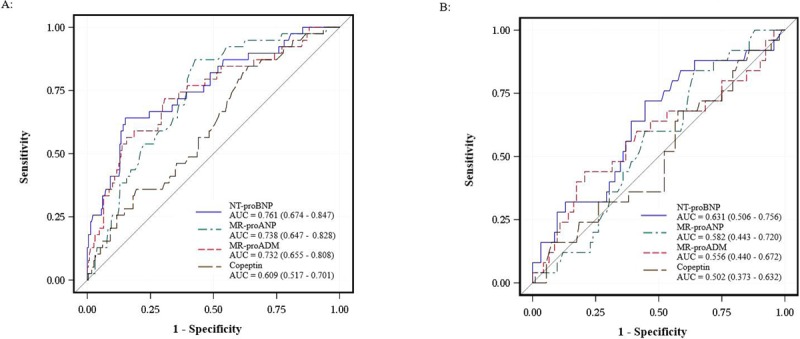
Receiver operating characteristics curves for the diagnosis of HF with NT-proBNP, MR-proANP, MR-proADM and copeptin among non-obese patients **(A)** and obese patients **(B)**.

ROC curves of the diagnostic ability for HF of the cardiac biomarkers, respectively, showed a poor performance in this outpatient population. The AUCs were higher in non-obese patients than obese patients for all cardiac biomarkers, however, 95%CI were overlapping (Figure 3). In non-obese patients the diagnostic accuracy was comparable between NT-proBNP, MR-proANP and MR-proADM (P > 0.05 for each comparison), whereas NT-proBNP (P = 0.002), MR-proANP (P = 0.009) and MR-proADM (P = 0.003) had a higher accuracy than Copeptin. In obese patients, no difference was found in the diagnostic accuracy between the cardiac biomarkers (P > 0.05 for all).

### Echocardiographic parameters, natriuretic peptides, and obesity

**Figure 4A–D:**
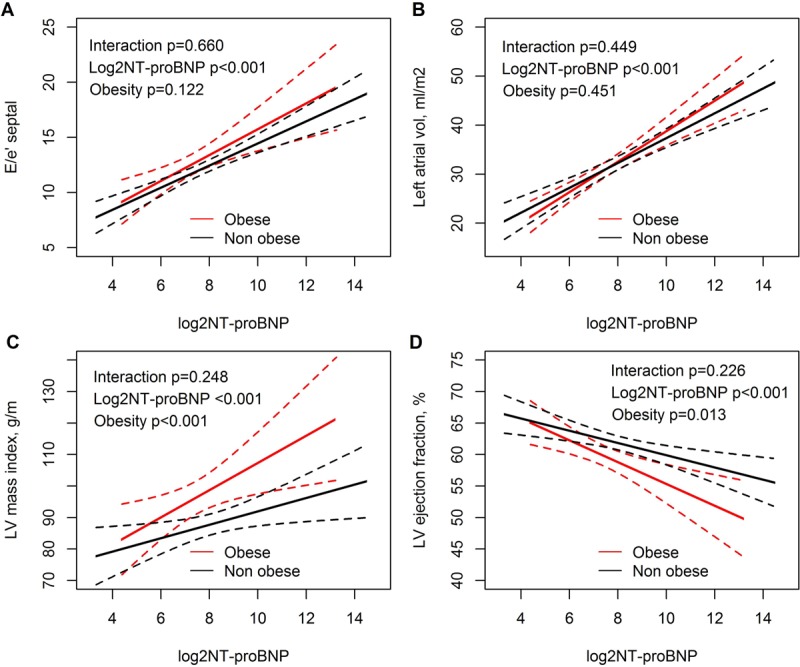
Correlations between echocardiographic parameters (dependent variables), and the plasma NT-proBNP concentrations, and obesity.

**Figure 5A–D:**
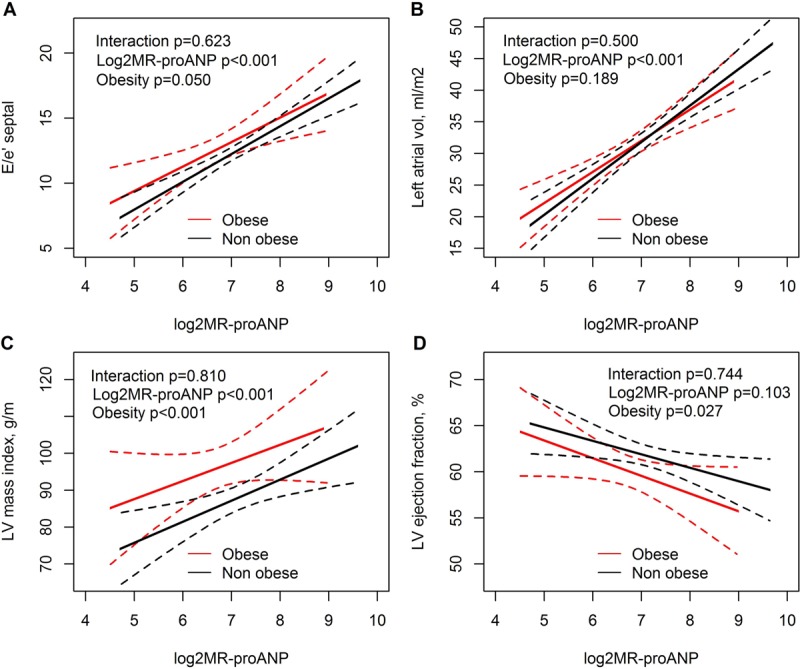
Correlations between echocardiographic parameters (dependent variables), and the plasma MR-proADM concentrations, and obesity.

The association between echocardiographic parameters, plasma NT-proBNP concentrations and obesity are shown in Figure 4A–D. Plasma NT-proBNP concentrations were positively associated with E/e’ septal, left atrial volume index, LV mass index, and inversely associated with LVEF. Additionally, in the same linear regression models, obesity was significantly associated with LV mass index and LVEF (supplemental Table 2). The association between echocardiographic parameters, plasma MR-proANP concentrations, and obesity are shown in Figure 5A–D. Plasma MR-proANP concentrations were positively associated with E/e’ septal, left atrial volume index, and LV mass index, but not with LVEF. In the same linear regression models, obesity was significantly associated with E/e’ septal, LV mass index and LVEF (supplemental Table 2).

## Discussion

The present study has two major findings: I) In obese patients, the diagnosis of HF was neither associated with increased plasma concentrations of NT-proBNP or MR-proANP nor with increased plasma concentrations of MR-proADM or copeptin, suggesting an overall impaired neurohormonal activation in obesity-related HF. II) The relationship between LV mass and LVEF and plasma concentrations of natriuretic peptides differ according to +/– obesity indicating that NP deficiency may play an important role in in the development of HF in obese patients already at a rather low BMI.

### Cardiac biomarkers and obesity

In this present study, obesity was accompanied by a natriuretic peptide deficiency. Previous studies have also demonstrated lower plasma concentrations of NT-proBNP in obese patients [11,12,28,29], as well as an increase in plasma concentrations of NT-proBNP in relation to an intended weight-loss [30]. This should be considered when plasma concentrations of NT-proBNP are used as a diagnostic tool in obese patients, where diagnostic challenges might be present, since poor physical conditions due to obesity can mimic symptoms of HF and thereby increase the risk of misdiagnosis of HF with accurate and normal plasma concentrations of NT-proBNP. However, the mechanism behind this lack of increase in the plasma concentrations of natriuretic peptides is unknown [13,30] and obesity-related HF may be a distinct phenotype characterized by an impaired glycose-metabolism and an increased inflammation, together with an altered hemodynamic. Thus, whether this is the case in the range of 30–35 of BMI is less well elucidated [16]. In the present study, the neurohormonal activation was also evaluated by plasma concentrations of MR-proADM and copeptin. Both were significantly higher in obese patients without HF, compared to non-obese patients without HF. Additionally, plasma concentrations of MR-proADM were significantly higher among the non-obese patients with HF compared to the non-obese patients without HF and not among obese patients with HF, suggesting that obesity influence the plasma concentrations of MR-proADM and copeptin in patients without HF and that obese patients with early stages of HF have an overall impaired neurohormonal response. A previous study has described higher plasma MR-proADM concentrations in obese patients [31]. In a population-based study, Sinning et al [29] reported similar results regarding plasma concentrations of NT-proBNP and MR-proANP, but in contrast to our results, plasma concentrations of MR-proADM were significantly higher in obese patients with HF compared to non-obese patients with HF. This discrepancy may be explained by differences in duration of HF between the studies and differences in the diagnosis of HF.

### Obesity, echocardiographic parameters, and natriuretic peptides

The echocardiographic findings in this present study were in accordance with previously reported structural and functional changes in obese patients [4,5]. Thus, obesity was correlated with both structural and functional changes in the heart and with natriuretic peptide deficiency. The association between plasma concentrations of NT-proBNP and MR-proANP and LV mass were shifted up-ward in obese patients, while the association between plasma concentrations of NT-proBNP and LVEF was shifted down-ward in obese patients indicating that for any given plasma concentration of the natriuretic peptides the obese patients have a higher LV mass and a lower LVEF. This may be explained by an increased afterload in obese patients, possibly due to increased arterial stiffness [32]. The association between plasma concentrations of the natriuretic peptides and left atrial volume index was not influenced by obesity. Left atrial remodelling reflects the burden of increased pressure and volume over time, but may be affected by other conditions than obesity e.g. atrial fibrillation and age [26]. E/e’ is a surrogate marker of LV filling pressure during certain circumstances [26]. Our findings suggest, that for any given value of plasma NT-proBNP concentrations, the LV filling pressure is equal in obese and non-obese patients, whereas any given value of the plasma MR-proANP concentration reflects a higher LV filling pressure in obese, compared to non-obese. The discrepancy between NT-proBNP and MR-proANP may reflect a type I error or that MR-proANP is closer associated with LV filling pressures than NT-proBNP [16].

### Diagnosis of HF in obese patients

Obesity mimics the symptoms of HF and thereby augments the challenge of diagnosing HF in obese patients. Dyspnoea may arise from a lower exercise capacity in obese patients [16]. The increased blood volume in obese patients predisposes to peripheral edema [7]. The clinical assessment of HF may be complicated by obesity and the image quality of the echocardiography in clinical practice may also be lower in obese patients. Accordingly, we observed a higher prevalence of patient-reported symptoms of HF and clinical evaluated signs of HF in obese patients without HF, compared to non-obese patients without HF. However, these patients did not fulfil the diagnostic criteria for HF, despite that symptoms and signs of HF were both present in a few patients. The prevalence of an abnormal echocardiography was comparable in obese and non-obese patients. Cardio-pulmonary exercise testing is a valuable test for discrimination cardiac- and non-cardiac dyspnoea [33]. Thus, in the present study, cardiopulmonary exercise testing may have reduced the risk of either a type 1 error (obesity associated edema and dyspnea with normal filling pressures) or a type 2 error (increased filling pressures during exercise despite lack of symptoms at rest).

In the 2016 guidelines on diagnosis and treatment of HF from the European Society of Cardiology plasma concentrations of NT-proBNP >125 ng/L were introduced as diagnostic criteria for all sub-types of HF [18]. Since plasma concentrations of natriuretic peptides are lower in obese patients, the diagnostic value for HF may be reduced in obese patients. Among the obese patients with HF, only 73.1% had plasma concentrations of NT-proBNP >125 ng/L, whereas, 89.7% of the non-obese patients with HF had plasma concentrations of NT-proBNP >125 ng/L. Accordingly, the risk of underestimating the prevalence of HFpEF and HFmrEF in obese patients is higher.

### Strengths and limitations

Some limitations of the study should be noted. The study participants were included from specialized units at the Herlev and Gentofte University Hospital, Denmark, according to specific in- and exclusion criteria. Results from this study, therefore, do not translate to the general population but instead brings insight to an elderly high-risk population. Nevertheless, these patients (elderly outpatients with one or more risk factor for HF, and a recent contact to the hospital) are not uncommon in the healthcare system, and the results, therefore, still seems clinically relevant.

The study was a cross-sectional study, without temporal information about the duration of obesity, weight gain or weight loss, the duration of symptoms or clinical signs of HF, and without repeated measurements of the cardiac biomarkers. Accordingly, we are not able to evaluate the effect of the duration of obesity, the effect of changes in BMI over time, or to evaluate causality between the impaired neurohormonal response and obesity. We used BMI to determine the obesity status of the patients. BMI is a surrogate measure of body fat, it is an established risk factor for HF with high reproducibility and easily accessible. However, BMI has some limitations that should be considered e.g. it does not account for muscle mass, and fat distribution [34]. Accordingly, other measures of body composition such as muscle mass, body fat percentage, hip-waist circumference could be important to explore in the future, as they may contribute to a better understanding of the mechanisms behind the natriuretic peptide deficiency in obese patients, and maybe even the obesity-paradox in HF.

The diagnosis of HF is oftentimes difficult in obese patients, with a risk of misclassification. To ensure that patient-reported symptoms of HF were indeed cardiac, we required the patients to present with both signs of dyspnoea (orthopnea or dyspnea) and edema (peripheral edema or treatment with loop diuretics). Clinical signs were based on clinical judgment and assessment of the NYHA functional class. The echocardiographic analyses were without systematic missing parameters, and analyses were validated by a trained physician. Baseline characteristics, echocardiographic parameters, and biochemical results in the present study were in accordance with findings in prior studies of obese patients with HF. Furthermore, no unexpected differences were encountered between the obese and non-obese patients with HF, suggesting a reliable diagnosis of HF among obese patients.

The cardiac biomarkers were tested using commercially available assays. However, test variability should be considered in the evaluations of biomarkers. Prior studies have reported substantial intra-individual variability in plasma concentrations of NT-proBNP e.g. age, sex, BMI [35]. In the present study we examined the influence of obesity on the plasma concentrations of specific cardiac biomarkers associated with heart HF, however, we did not calculate intra-individual variability of the plasma concentrations. In the statistical analysis, we adjusted for several covariates, known confounders from the Framingham HF risk study and other relevant parameters, but there is still a risk of unmeasured confounding.

## Conclusion

Obesity-related HF was associated with natriuretic peptide deficiency and an overall impaired neurohormonal activation in elderly outpatients with risk factors for HF. Structural and functional changes of the myocardium in early stages of obesity-related HF seemed close correlated to increased afterload rather than volume overload.

## Clinical perspectives

Obesity-related HF are characterized by a lower age, higher prevalence of diabetes and lower prevalence of atrial fibrillation, compared to non-obese patients with HF.

The plasma concentrations of NT-proBNP and MR-proANP are lower in obese patients, this should be considered in clinical practice when interpreting these biomarkers.

Patient-reported symptoms and clinical signs of HF are more prevalent among obese patients. This complicates the diagnosis of HF in obese patients, alongside with the natriuretic peptide deficiency. Additional methods may be needed for the evaluation of cardiac performance in obese patients e.g. cardiopulmonary exercise testing or invasive hemodynamic testing.

## Translational outlook

Future studies should seek to increase knowledge of the mechanisms behind obesity-related HF, e.g. by investigating the hemodynamic changes in the early stages of HF in obese patients.

Further, these data suggest an over-all impaired neurohormonal activation in obesity-related HF. Accordingly, the conventional treatment strategy of HF, with modulation of the neurohormonal activation, may not have the same effect in obese patients with HF, and instead, novel treatment strategies should be considered e.g. intended weight loss, mineralocorticoid receptor antagonist, sodium–glucose co-transporter-2 inhibitor, LCZ696. However, these concepts need to be tested in randomized controlled trials.

## Additonal Files

The additional files for this article can be found as follows:

10.5334/gh.776.s1 Supplemental Table 1. Logistic regression.

10.5334/gh.776.s2 Supplemental Table 2. Correlations between echocardiographic parameters and the NPs and obesity.

## Abbreviations

HF = heart failure

LV = left ventricle

MR-proADM = mid-regional pro-adrenomedullin

NT-proBNP = amino-terminal pro-B-type-natriuretic-peptide

MR-proANP = mid-regional pro-atrial-natriuretic-peptide

EF = ejection fraction

BMI = body mass index

PCWP = pulmonary capillary wedge pressure

BSA = body surface area

NYHA = New York Hear Association

LVEDV = left ventricle end-diastolic volume
