# Supplementary material for: Early Stages of Obesity-related Heart Failure Are Associated with Natriuretic Peptide Deficiency and an Overall Lack of Neurohormonal Activation: The Copenhagen Heart Failure Risk Study
Source: Glob Heart. 2020 Mar 25;15(1):25. doi: 10.5334/gh.776 (PMC7218759; doi:10.5334/gh.776)
Supplement: Supplemental Table 1. — Logistic regression. [file gh-15-1-776-s1.pdf]

Supplemental Table 1. Logistic regression. Association between BMI categories and heart failure in logistic regression models (outcome variable: heart failure).

	**Odds Ratio**<br>**95% Confidence Interval**	**Estimate**<br>**95% Confidence Interval**	**p-value**
Model 1			
**BMI 18.5-24.9**	1 (ref)	--	--
**BMI 25-29.9**	1.46 (0.69 – 3.07)	-0.130 (-0.518 – 0.257)	0.511
**BMI > 30**	3.15 (1.43 – 6.91)	0.638 (0.223 – 1.054)	0.003
Model 2			
**BMI 18.5-24.9**	1 (ref)	--	--
**BMI 25-29.9**	1.36 (0.63 – 2.91)	-0.186 (-0.582 – 0.211)	0.359
**BMI > 30**	3.21 (1.40 – 7.34)	0.676 (0.239 – 1.113)	0.003
Model 3			
**BMI 18.5-24.9**	1 (ref)	--	--
**BMI 25-29.9**	1.32 (0.61 – 2.87)	-0.190 (-0.589 – 0.209)	0.350
**BMI > 30**	3.10 (1.34 – 7.16)	0.660 (0.220 – 1.101)	0.003

Model 1: Age and gender (reference female)

Model 2: variables in model 1, Diabetes, Atrial fibrillation, Hypertension, Ischemic heart disease, eGFR<60ml/min/1.73m^2, Stroke

Model 3: variables in model 2, LDL cholesterol <2.6mmol/L, HDL cholesterol >1.2mmol/L women and >1.0mmol/L men, Lipoprotein A >50mg/dL
