# Supplementary material for: Early Stages of Obesity-related Heart Failure Are Associated with Natriuretic Peptide Deficiency and an Overall Lack of Neurohormonal Activation: The Copenhagen Heart Failure Risk Study
Source: Glob Heart. 2020 Mar 25;15(1):25. doi: 10.5334/gh.776 (PMC7218759; doi:10.5334/gh.776)
Supplement: Supplemental Table 2. — Correlations between echocardiographic parameters and the NPs and obesity. [file gh-15-1-776-s2.pdf]

Supplemental table 2. Correlations between echocardiographic parameters and the NPs and obesity.

Response variable were specific echocardiographic parameters in separate models. All models were adjusted for age, sex, eGFR and atrial fibrillation.

	**E/e'**	**LA volume index**	**LV mass index**	**LVEF**
		Model with Log_2 NT-proBNP		
**Log_2 NT-proBNP**	Estimate: 0.932<br>95%CI: 0.616 - 1.248<br>p<0.001	Estimate: 2.576<br>95%CI: 1.852 - 3.300<br>p<0.001	Estimate: 5.088<br>95%CI: 3.294 - 6.881<br>p<0.001	Estimate: -1.149<br>95%CI: -1.778 – -0.520<br>p<0.001
**Obesity**	Estimate: 0.767<br>95%CI: -0.206 - 1.741<br>p=0.122	Estimate: 0.897<br>95%CI: -1.441 - 3.234<br>p=0.451	Estimate: 10.450<br>95%CI: 4.639 - 16.261<br>p<0.001	Estimate: -2.556<br>95%CI: -4.576 - -0.536<br>p=0.013
		Model with Log_2 MR-proANP		
**Log_2 MR-proANP**	Estimate 1.817<br>95%CI: 1.146 - 2.487<br>p<0.001	Estimate: 6.312<br>95%CI: 4.775 - 7.849<br>p<0.001	Estimate: 10.682<br>95%CI: 6.798 - 14.567<br>p<0.001	Estimate: -1.141<br>95%CI: -2.514 - 0.232<br>p=0.103
**Obesity**	Estimate: 0.988<br>95%CI: -0.001 - 1.976<br>p=0.050	Estimate: 1.548<br>95%CI: -0.763 - 3.858<br>p=0.189	Estimate: 11.337<br>95%CI: 5.465 - 17.209<br>p<0.001	Estimate: -2.318<br>95%CI: -4.375 – -0.262<br>p=0.027

Abbreviations: NP= natriuretic peptide; LV=left ventricle; LA= left atrial; EF= Ejection fraction
